# Cre toxicity in mouse models of cardiovascular physiology and disease

**DOI:** 10.1038/s44161-022-00125-6

**Published:** 2022-09-09

**Authors:** Victoria S. Rashbrook, James T. Brash, Christiana Ruhrberg

**Affiliations:** 1 UCL Institute of Ophthalmology, University College London, 11-43 Bath Street, London EC1V 9EL, UK

## Abstract

The Cre-LoxP system provides a widely used method for studying gene requirements in the mouse as the main mammalian genetic model organism. To define the molecular and cellular mechanisms that underlie cardiovascular development, function and disease, various mouse strains have been engineered that allow Cre-LoxP-mediated gene targeting within specific cell types of the cardiovascular system. Despite the usefulness of this system, evidence is accumulating that Cre activity can have toxic effects in cells, independently of its ability to recombine pairs of engineered LoxP sites in target genes. Here, we have gathered published evidence for Cre toxicity in cells and tissues relevant to cardiovascular biology and provide an overview of mechanisms proposed to underlie Cre toxicity. Based on this knowledge, we propose that each study utilising the Cre-LoxP system to investigate gene function in the cardiovascular system should incorporate appropriate controls to account for Cre toxicity.

## Introduction

**Figure 1:**
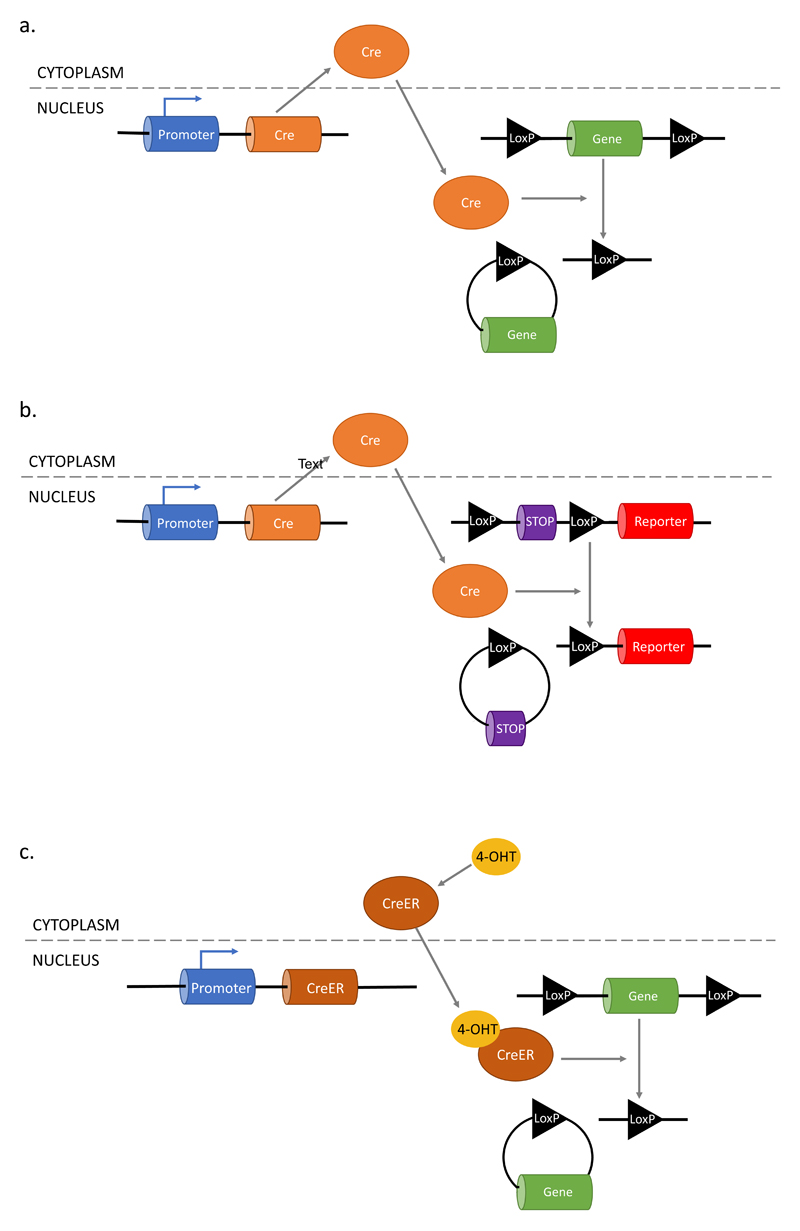
Cre-LoxP mediated recombination of target genes. Following translocation to the nucleus, Cre recombines loxP sites that have been engineered into the mouse genome, thereby excising the intervening sequences. **a,** The Cre–LoxP system can be used to delete a critical exon in a floxed gene. **b,** The Cre–LoxP system can delete a floxed stop codon to activate the expression of a reporter gene, which allows monitoring of Cre activity and genetic lineage tracing. **c,** The CreER fusion protein is retained in the cytoplasm until 4- hydroxytamoxifen (OHT) binding induces nuclear translocation, termed CreER activation, for example to remove a stop codon in front of a reporter.

Conditional mutagenesis with the Cre–LoxP system has revolutionised mouse genetics^1,2^. For this method, the bacteriophage recombinase Cre is expressed from a transgene or after knock-in into an endogenous genomic locus in the mouse to recombine genomic regions that are engineered to be ‘flanked by LoxP’ recognition sites, also known as ‘floxing’^1,2^. Whereas floxing a critical exon allows gene silencing (Fig. 1A), floxing a stop codon upstream of a reporter allows genetic lineage tracing of Cre-activated cells and their progeny when the reporter cassette is placed into a constitutively active locus or into transgene with a strong promoter (Fig. 1B)^3–10^. For recombination efficiency, a suitable endogenous promoter must be selected to drive Cre expression. For example, nearly ubiquitous Cre expression can be achieved with the chicken beta actin promoter (also known as CAG), such as in the Tg(CAG-cre)13Miya transgene^11^. However, ubiquitously deleting genes with essential developmental functions might cause embryonic lethality or cause complex phenotypes for genes that are expressed in multiple cell types^12^. Accordingly, gene ablation is often spatially restricted with the use of cell type-specific promotors to drive Cre expression. For example, using the *Cdh5* promoter restricts Cre expression to vascular endothelial cells^13^.

**Figure 2:**
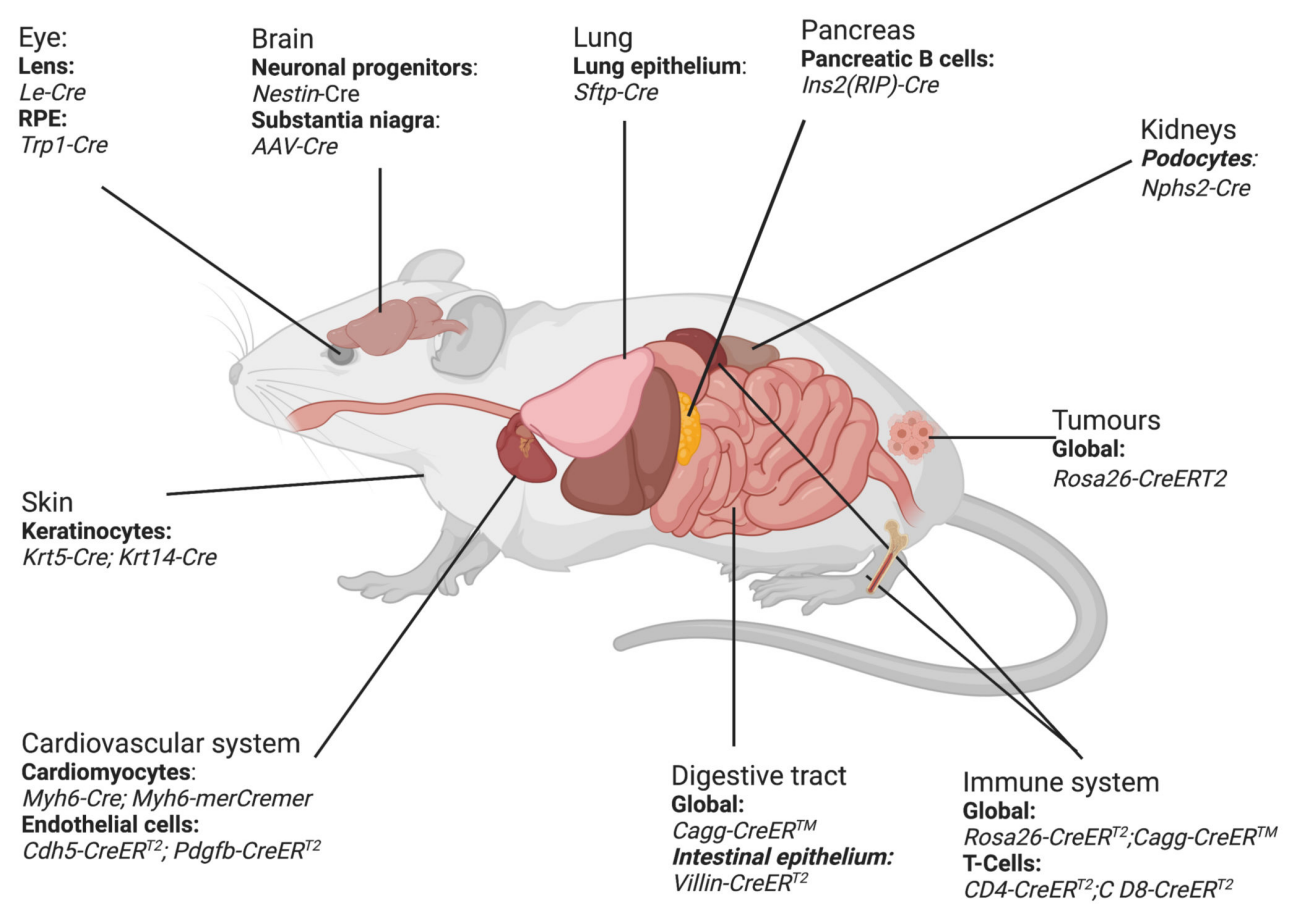
Organs affected by Cre toxicity. Schematic representation of mouse organs known to be affected by Cre toxicity, including affected cell types and toxicity inducing Cre and CreER models. For exact transgene nomenclature, see main text. RPE, retinal pigment epithelium.

Temporal control of gene deletion can be achieved by fusing Cre to the oestrogen receptor (ER) ligand binding domain^14^. The ER domain retains the fusion protein in the cytoplasm until ligand binding induces nuclear translocation as a prerequisite to targeting floxed genes (Fig. 1C). A range of CreER fusion constructs are used for inducible gene deletion. CreER^T^ is a human ER variant with a single mutation that confers selectivity to the tamoxifen metabolite 4-hydroxytamoxifen (4-OHT) over endogenous 17β-oestradiol, and CreER^T^-expressing mice provided proof that inducible gene deletion was achievable with high specificity *in vivo^15,16^*. Subsequently, the CreER^T1^ and CreER^T2^ constructs with further mutations were engineered to increase sensitivity^17^. Alternative CreER fusions include CreER™, which utilises a murine ER domain with an analogous mutation to human CreER^T^, and MerCreMer, in which Cre is bound to two mutant murine ER ligand binding domains^14,18,19^. When CreER expression is driven by cell type-specific promoters, both spatial and temporal control can be achieved^20,21^. Accordingly, the Cre–LoxP system is widely used to define the molecular and cellular mechanisms that underpin organ development, adult physiology or disease. However, a growing number of studies have reported that Cre expression or CreER activity causes toxicity in multiple organ systems, including in the cardiovascular system^22–31^(Fig. 2). Presently, cardiovascular researchers rarely consider this knowledge when seeking to improve their experimental design.

Here, we provide an overview of published Cre and CreER toxicity studies relevant to the cardiovascular system, describe known molecular and cellular mechanisms that underlie toxicity, and discuss the potential differences between Cre and CreER. Based on the knowledge gathered, we argue that future Cre–LoxP-based studies should incorporate appropriate controls to discover, and account for, cellular and organism-wide phenotypes caused by Cre/CreER toxicity. Considering this recommendation will ensure that the mouse continues to provide a reliable genetic model organism for mechanistic studies of cardiovascular development, function and disease.

### Cre toxicity in the cardiovascular system

Many studies have used Cre–LoxP technology to identify cell lineages giving rise to the heart or blood vessels or to ablate genes in these cell lineages. Although most cardiovascular studies have not reported toxicity, others identified toxic effects in several cell types that comprise the cardiovascular system or that interact with it.

### Cre toxicity in cardiomyocytes

*Myh6* encodes one of two myosin heavy chain proteins for cardiac contraction^32^, and the *Myh6* promoter has been used to express Cre or CreER in cardiomyocytes. Tg(Myh6-cre)2182Mds drives constitutive Cre expression (MGI: 2386742)^3^ and Tg(Myh6-cre/Esr1*)1Jmk drives inducible Mer-Cre-Mer expression (MGI:3050453)^33^. Expressing either Cre or activated CreER in cardiomyocytes causes cardiac dysfunction, with some sex and age specific differences^34–38^.

One study found that *Myh6-Cre* male mice have a reduced heart rate and irregular ejection fraction, which increased at 3 months of age compared with Cre-negative controls, and that *Myh6-Cre* female mice had similar defects at 6 months but not 3 months of age^34^. The heart tissue of both sexes reactivates foetal genes indicative of cardiac damage, such as *Anp* and *Bnp*^34^. Another study reported increased cardiac fibrosis and cardiomyocyte size in *Myh6-Cre* mice compared with Cre-negative controls as well as decreased body weight and survival^35^. These findings demonstrate cardiomyocyte vulnerability to Cre, with unidentified sex-dependent modifiers.

Like *Myh6-Cre* males, *Myh6-MerCreMer* males treated with tamoxifen at 3 months of age have a decreased ejection fraction and left atrial dilation compared with untreated controls^36^. Given that cardiac fibrosis in *Myh6-MerCreMer* mice 1, 6 or 7 weeks after treatment occurred with high, but not low tamoxifen doses^37,38^, CreER toxicity appears to be dose-dependent. Although cardiac defects were apparent 10 days after CreER activation with high tamoxifen doses, they began to recover by 28 days after induction^36^. This finding suggests that transient Cre activity allows for partial functional recovery from cardiotoxicity, although the specific mechanism underlying recovery remains unknown. Notably, recovery did not occur in tamoxifen-treated *Myh6-MerCreMer* males also carrying floxed *Pi3ka* alleles, which suggests that PI3Ka protects from CreER toxicity^36^.

### Cre toxicity in vascular endothelial cells

Several Cre transgenes targeting vascular endothelial cells incorporate the *Tek (Tie2), Cdh5* or *Pdgfb* promoters. The *Tie2* promoter is used in Tg(Tek-cre)1Ywa (MGI:2450311)^39^ and Tg(Tek-cre)5326Sato (MGI:2445474)^40^, which are both active in many vascular beds and are known as *Tie2*-Cre. Commonly used Cre transgenes utilising the endothelial-ubiquitous *Cdh5* promoter include Tg(Cdh5-cre/ERT2)1Rha (MGI:3848982) and Tg(Cdh5-cre/ERT2)Ykub (MGI:5705396)^41,42^. The *Pdgfb* promoter has been incorporated into Tg(Pdgfb-icre/ERT2)1Frut (MGI:3793852) to drive CreER expression in many vascular beds, especially in the brain and retina^43^. A subset of these promotors has been examined for endothelial toxicity^44,45^

**Figure 3:**
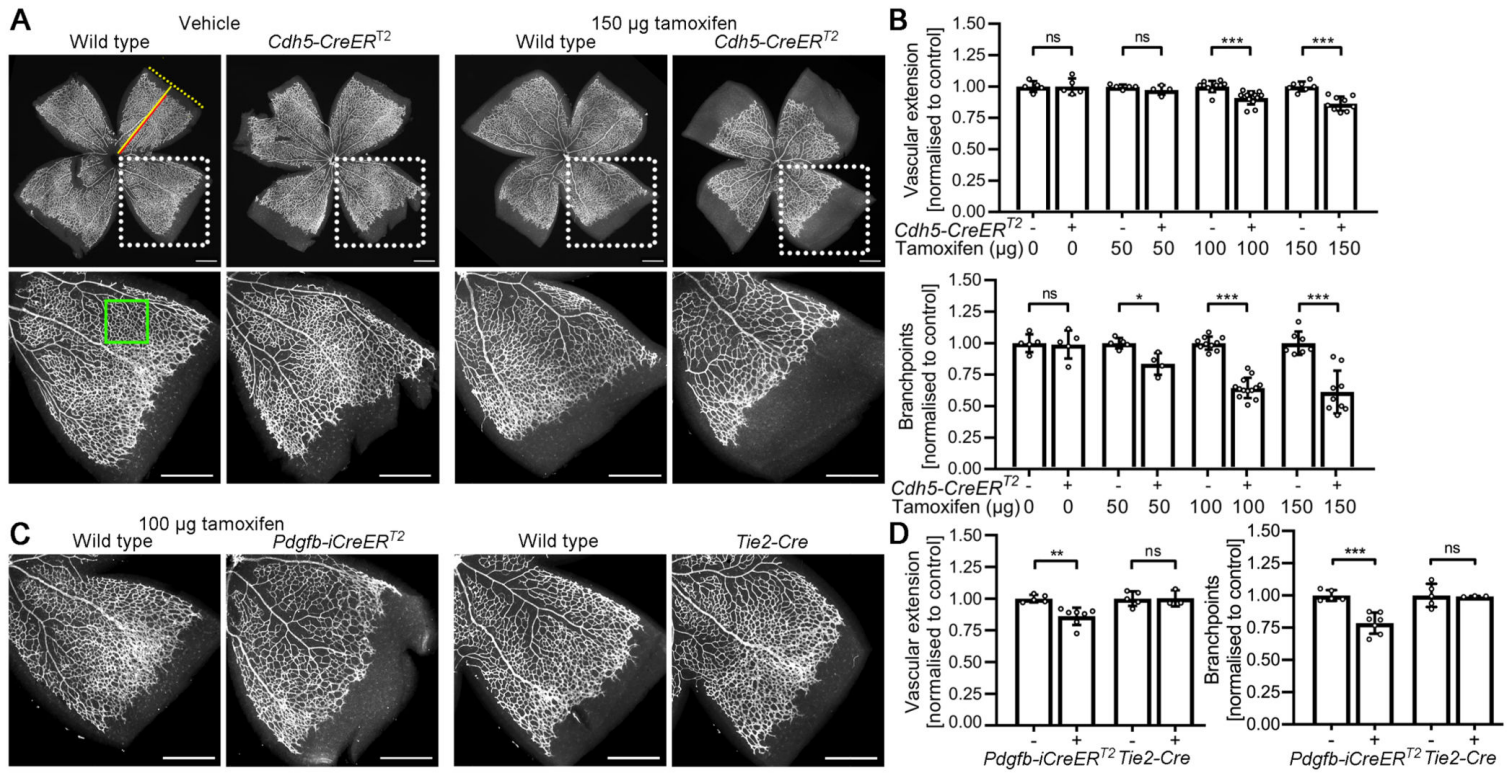
Endothelial CreER^T2^ activation impairs retinal angiogenesis. Flat mounted perinatal day (P)7 retinas were stained with the vascular endothelial marker isolectin (IB4) and fluorescent streptavidin. **a** and **b** *Cdh5*-CreERT2–expressing and wildtype littermates were injected at P2 and P4 with 25 μL peanut oil containing 0, 50, 100, or 150 μg tamoxifen. **a,** Micrographs and **b**, quantification of vascular extension and branch density. Dotted boxes indicate areas shown at higher magnification. Red and yellow lines indicate vascular extension and retinal radius. The green box indicates a representative area analysed for vascular branch density. Scale bars: 500 μm. (c) and (d), *Pdgfb*-iCreERT2-expressing and wildtype littermates were injected at P2 and P4 with 25 μL peanut oil containing 100 pg tamoxifen. Tie2–Cre litters were not injected. **c**, Micrographs and **d**, quantification of vascular extension and branch density. Data are presented as mean±SD fold change relative to littermate controls; each data point represents the average of several retinal leaflets. *Cdh5*–CreERT2 experiments: controls n=5 (0 μg), n=5 (50 μg), n=10 (100 μg), n=7 (150 μg); CreERT2 n=5 (0 μg), n=4 (50 μg), n=13 (100 μg), n=9 (150 μg); *Pdgfb*–iCreERT2 experiments: controls n=5, CreERT2 n=7; *Tie2–Cre* experiments: controls n=5, *Tie2*–Cre n=3. Two-way ANOVA with Holm-Sidak multiple comparison test, non-significant (ns), P>0.05; *P<0.05; **P<0.01, ***P<0.001. Figure and corresponding legend adapted with permission from the publisher^106^.

Tamoxifen-treated mice expressing CreER^T2^ under the control of the *Cdh5* (MGI:5705396) or *Pdgfb* (MGI:3793852) promoters have impaired retinal angiogenesis on postnatal day (P7) when compared with tamoxifen-treated, CreER^T2^-negative littermates (Fig. 3A)^45,46^. Specifically, vascular outgrowth across the retina and vascular branching density are reduced (Fig. 3B,C)^45,46^. By contrast, retinal angiogenesis is not affected in vehicle-injected mice expressing CreER^T2^ or vehicle-treated CreER^T2^-negative control mice^45^, suggesting that CreER^T2^ toxicity depends on 4-OHT-induced nuclear localisation. Analogous to observations with cardiomyocyte toxicity, the severity of CreER-induced retinal angiogenesis defects correlates with tamoxifen dose^45^. Of note, retinal angiogenesis defects occurred without a general developmental or growth delay, indicated by similar retinal radius and whole body weight in CreER^T2^-expressing and CreER^T2^-negative P7 littermates at the highest tamoxifen dose examined^45^.

Tamoxifen-induced CreER toxicity with two different transgenes, using different promoters and integrated randomly into the genome, implies that CreER toxicity in endothelial cells is not a specific feature of any individual transgenes, but caused by CreER activation. Accordingly, investigating whether other commonly used CreER transgenes cause toxicity in retinal angiogenesis is pertinent, because the mouse perinatal retina is the most widely used model to study the molecular and cellular mechanisms of angiogenesis^47,48,49^.

Toxicity phenotypes might have been accounted for in some studies by including appropriate controls, even when this was not specifically stated^50,51^, whereas other studies might not have considered that described phenotypes were confounded by CreER toxicity. Of note, toxicity is not a specific feature of tamoxifen, because 4-OHT also induces toxicity^45^. Thus, we recommend that published studies reporting retinal angiogenesis defects after CreER-mediated recombination of floxed endothelial genes are retrospectively evaluated to consider whether observed defects may be partially or wholly attributable to CreER toxicity. We further recommend that future studies should include appropriate CreER toxicity controls (see below). Particularly, it would we helpful to establish whether specific combinations of transgene type and dosing schedules affect radial expansion or branching of retinal vasculature, independently of the floxed gene.

In contrast to tamoxifen-activated *Cdh5-CreER^T2^* (MGI:5705396) and *Pdgfb-CreER^T2^* (MGI:3793852), the constitutive *Tie2-Cre* (MGI:2450311) does not cause obvious retinal angiogenesis defects (Fig. 3)^45^. This finding is surprising, because *Tie2-Cre* is active from early embryogenesis onwards and throughout postnatal development^39^. The lack of postnatal angiogenesis defects in *Tie2-Cre* mice may indicate that the native Cre is less toxic for endothelial cells than 4-OHT-bound CreER, but the molecular mechanisms underlying differential toxicity remain to be determined and compared. Alternatively, constitutive Cre expression might be toxic to endothelial cells, as observed for cardiomyocytes, but Cre-induced endothelial toxicity is less readily observed. For example, Cre-induced vascular defects might be transient, if the endothelial cell population could adapt to the Cre insult over time.

Even if Cre and activated CreER were equally toxic when expressed at similar levels and under similar circumstances, toxicity differences might arise with different transgene expression levels, which themselves could be due to differences in promoter activity or transgene copy number. For example, *Tie2-Cre* (MGI:2450311) was present in 2-20 copies in the initial study^39^, but a commonly used sub-strain, distributed through JAX Laboratories, only carries 3-4 transgene copies (https://www.jax.org/strain/008863). Although it remains unknown to what extent copy number variation impacts Cre/CreER toxicity in the cardiovascular system, it has been described for the neural and immune systems (see below).

In summary, work to date suggests that more detailed toxicity analysis is warranted for CreER mouse lines used in angiogenesis research, including lines not yet tested for toxicity. Future work should examine whether CreER toxicity-induced retinal angiogenesis defects resolve over time and whether endothelial CreER toxicity impairs angiogenesis in other tissues.

### Cre toxicity in blood cells

The vasculature transports blood cells and provides a platform for immune surveillance; in turn, the immune system modulates cardiovascular physiology and disease, for example, in angiogenesis^52^, arteriogenesis^53^ and inflammation^54^, including during atherosclerosis^54^. Therefore, cardiovascular researchers should consider that Cre/CreER toxicity has been observed in the haematopoietic and immune systems. For example, activating the ubiquitously expressed *Rosa26*-CreER^T2^ transgene during embryogenesis reduces erythrocyte numbers and decreases embryo size^55^. Moreover, *Rosa26*-CreER^T2^ activation in adulthood causes thymic atrophy and decreases bone marrow cellularity, with an increased proportion of bone marrow cells showing chromosomal aberrations^55^. *Rosa26*-CreER^T2^ activation also decreases CD8^+^ T-cell number and clonal expansion^56^. Additionally, activating Tg(Cd4-cre/ERT2)11Gnri (MGI:5464279) in T-cells expressing the CD4 glycoprotein reduces the number of activated T-cells^56,57^. If hematopoietic cells are particularly sensitive to CreER toxicity, then using transgenes active in these cells might cause compounding phenotypes in cardiovascular studies via altered oxygenation or cardiovascular inflammation.

### Cre toxicity in cell types that regulate cardiovascular function

Hyperglycaemia owing to impaired insulin secretion causes endothelial inflammation, hyperpermeability and cell death^58–61^. *Ins2* encodes the insulin 2 protein, which is pivotal for glucose homeostasis^62^. Tg(Ins2-cre)25Mgn (MGI:2176227) mice expressing Cre in pancreatic β-cells from the *Ins2* promoter have decreased blood insulin levels following glucose injection, even in the absence of floxed target genes^63^. Whereas young Tg(Ins2-cre)25Mgn mice have decreased β-cell mass, β-cell mass increases with age compared with wild type controls, probably owing to compensatory hyperproliferation^64^. Whether reduced insulin levels due to Cre toxicity causes cardiovascular phenotypes remains to be determined.

### Mechanisms of Cre and CreER toxicity

The molecular and cellular mechanisms of Cre/CreER toxicity have been studied in diverse cell types, although not always *in vivo*, and, with few exceptions, not explicitly in cardiovascular cell types. Therefore, we review the literature of Cre/CreER toxicity mechanisms from studies of other cell types and their host organs to argue that such mechanisms should also be examined when Cre or CreER are used to investigate cardiovascular development and function. Where available, we will explicitly refer to knowledge for cardiovascular cell types.

### DNA damage and chromosomal abnormalities

DNA damage following Cre expression or CreER activation is commonly reported in Cre toxicity studies. For instance, mouse embryonic fibroblasts (MEFs) expressing Cre or 4-OHT-activated CreER^T2^ have more chromosomal abnormalities than controls^65,66^. Further, 4-OHT-treated MEFs or mouse keratinocytes expressing activated CreER^T2^ have more cells with polyploid nuclei when compared to untreated controls or cells expressing endonuclease-deficient CreER^T2 31,65^. Expression of gamma-H2AX, a DNA damage marker, is upregulated when CreER^T2^ is activated in keratinocytes^31^. *In vivo*, the gastric epithelium of tamoxifen-treated CAG-CreER™ mice has increased expression of the DNA damage markers *Ddit3* and *Gadd45a* compared with Cre-negative controls^29^. In the cardiovascular system, the *Myh6-Cre* myocardium has increased levels of the DNA damage markers PARP and BAX compared with Cre-negative myocardium^34^. Analogous studies are outstanding for other cardiovascular cell types and cardiovascular-relevant Cre/CreER lines to determine whether DNA damage is a common response to Cre expression or CreER activation.

DNA damage in the studies described above is not explained by cleavage of endogenous *LoxP* consensus sites, because they are absent from the mouse and human genomes. However, several studies identified ‘pseudo-LoxP sites’, defined as genomic sequences with LoxP homology that may be recognised by Cre^34,67,68^. *In silico* mapping of the mouse and human genomes identified 123 sites with homology to the LoxP consensus sequence with 4 or fewer mismatches^67^. Among these pseudo-LoxP sites, one site had an *in vitro* cleavage efficiency similar to wildtype LoxP^67^.

The presence of genomic pseudo-*LoxP* sites raises the possibility that Cre can cleave pseudo-*LoxP* sites to attempt recombination, but the absence of a neighbouring *LoxP* site then prevents recombination, thus leaving a DNA break requiring repair. Consistent with this idea, previous work linked non-homologous end joining to Cre toxicity, with cellular defects dependent on Cre endonuclease activity^65^. Consistent with Cre-induced DNA damage at pseudo-LoxP sites, the *Mdr1b* gene that harbours one pseudo-LoxP site was expressed at lower levels in mice with activated Villin-CreER^T2^ (MGI:3053826) compared with control mice^28,67^. Another study extended the number of potential pseudo-LoxP sites in the mouse genome to 619; 227 of these are located within known genes, including 55 genes expressed in the myocardium^34^. 27 of these 55 genes were further analysed, and 26% were found to be differentially expressed in hearts expressing Cre^34^.

**Figure 4:**
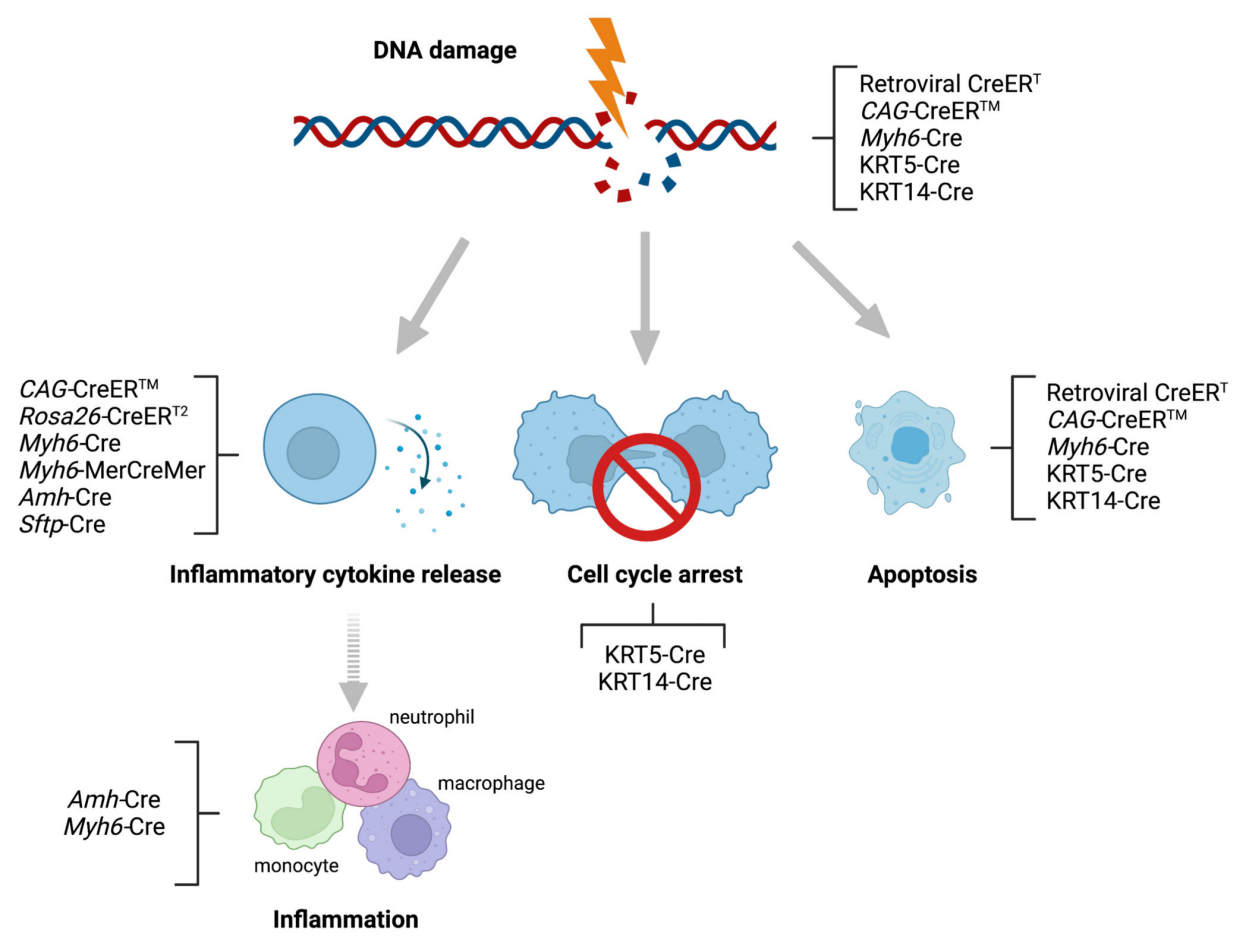
Cre/CreER-induced toxicity carries hallmarks of known cellular responses to DNA damage. Although DNA damage normally induces repair mechanisms to maintain cell viability, ineffective repair in response to Cre/CreER toxicity can trigger three different damage responses, namely cell inflammatory cytokine release, cycle arrest or apoptosis (indicated by arrows). These cellular responses have been observed in studies using retroviral CreER and the indicated Cre or CreER transgenes (responses may not be mutually exclusive). It is likely that Cre/CreER toxicity-induced cytokine release causes sterile inflammation, typically mediated by monocytes, macrophages and neutrophils (indicated with dashed arrows). Cre/CreER toxicity-induced cell cycle arrest and apoptosis might also cause sterile inflammation.

DNA damage induces three different signalling pathways to reduce the proliferation of damaged cells, all of which have been linked to p53 activation (Fig. 4)^69^. In the first pathway, double-stranded DNA breaks activate the kinase ATM, which stabilises p53 and induces p53-dependent DNA repair^69^. The second pathway causes cell cycle arrest in the G1/G2 phases of mitosis, mediated partly through p53 and gamma-H2AX^69^. The third pathway involves p53-dependent apoptosis^69^. Consistent with a p53-mediated DNA damage response, gastric epithelial cells from tamoxifen-induced CAG-CreER™ mice^29^ and Sertoli cells expressing *Amh-Cre* have increased nuclear levels of the p53-binding protein 53BP1 compared with control cells^70^. Furthermore, limb-skeletal shortening in *Fabp4-Cre* mice is attenuated by p53 ablation^71^. This p53-mediated exacerbation of Cre toxicity might be explained by increased apoptosis after failed DNA repair (Fig. 4).

In the cardiovascular system, increased p53 expression is observed in cardiomyocytes from *Myh6-Cre* mice compared with Cre-negative controls, although it remains unknown whether p53 promotes or ameliorates Cre toxicity in cardiomyocytes^72,73^. Therefore, it would be important to investigate whether p53 is also activated in response to DNA damage in endothelial cells expressing Cre or activated CreER. Whereas acute p53 upregulation might cause apoptosis, chronic p53 upregulation with constitutive Cre expression (for example, *Tie2*-Cre) might mitigate toxicity through activating DNA repair mechanisms (Fig. 4), thereby enabling a stronger adaptive response when compared with acute CreER recombinase activation (for example, *Cdh5*-CreER^T2^).

Taken together, we propose investigating whether Cre/CreER-induced DNA damage in cardiovascular cell types induces DNA repair mechanisms that can mitigate toxicity, either via successful DNA repair or cell cycle arrest and/or apoptosis to remove irreparably damaged cells (Fig. 4).

### Impaired cell proliferation and apoptosis

Consistent with the consequences of DNA damage, impaired cell growth has been reported in cells expressing Cre or activated CreER. For example, MEF cultures expressing a Cre–GFP fusion protein grow slower than control cultures^65^. Similarly, cultures of immortalised CV-1 and COS cells infected with a lentivirus that incorporates Cre into genomic DNA grow slower than control cells lacking Cre^74^. CreER^T^ activity also impairs the expansion of MEFs and NIH-3T3, COS-7, HeLa and U2OS cells^65^. Defective growth of MEF cultures expressing CreER^T^ is 4-OHT dose-dependent, and endonuclease-null CreER^T^ does not cause a growth defect^65^, thereby demonstrating that toxicity is caused by the 4-OHT-induced CreER recombinase activity. Therefore, *in vitro* studies support the hypothesis that DNA damage underpins Cre/CreER toxicity.

In principle, defective growth might be due to impaired proliferation, increased apoptosis, or a combination of both. Consistent with p53-induced cell cycle arrest or apoptosis downstream of DNA damage, CreER^T^-expressing MEFs are over-represented in the sub-G1 phase and under-represented at the G0/G1 and S phase checkpoints after 4-OHT treatment compared with untreated controls^65^. Moreover, mouse keratinocytes with activated CreER^T2^ have decreased nuclear localisation of cyclin B1, indicating reduced cell cycle propagation^31^. Decreased cell viability has instead been reported for Cre-expressing HeLa cells^75^. *In vivo*, increased apoptosis has been described in gastric epithelial cells after CAG-CreER™ activation^29^, in Sertoli cells expressing *Amh*-Cre^70^ and in lung epithelial cells expressing *Sftp-Cre^26^*. Moreover, embryos expressing *Rosa26*-CreER^T2^ have increased cell death compared with CreER-negative controls after maternal tamoxifen treatment, and cell death incidence positively correlates with tamoxifen dose^76^. In the cardiovascular system, TUNEL staining to detect apoptosis is significantly increased in cardiomyocytes of *Myh6-Cre* and tamoxifen-treated *Myh6*-merCremer mice compared with Cre/CreER-negative controls^34,38^. Together, these observations suggest that apoptosis contributes to Cre/CreER-induced toxicity *in vitro* and *in vivo*, including in the cardiovascular system.

### Altered inflammatory and metabolic signalling

Expression of Cre or activated CreER has been associated with dysregulated cell signalling (Table 1). For example, global phosphorylation levels are decreased in MEFs expressing Cre or activated CreER^T2^, which was attributed to impaired protein kinase A (PKA) signalling^77^. Given that PKA activation regulates inflammation and metabolism^78^, it is interesting that Cre and activated CreER affect gene expression in relevant signalling pathways^27,34,70^. For example, CreER^T^ activation in MEFs, mouse bone-marrow derived macrophages or human epithelial cells upregulates signalling from the inflammatory cytokine IFN-1^79^. Moreover, Sertoli cells expressing *Amh*-Cre increase expression of the cytokines IL1 and IL6 compared with Cre-negative littermates241. In the cardiovascular system, *Myh6-Cre* mice increase expression of IL6 and IL1β compared with Cre negative controls^34^.

Sertoli cells in *Amh*-Cre mice increase the expression of peroxisomal lipid metabolism genes and antioxidant enzymes, accompanied by a perturbed balance of sirtuins^70^, which modulate levels of histone acetylation and are targets of PKA signalling^80^. Specifically, Sertoli cells expressing *Amh*-Cre downregulate SIRT1 transcripts but upregulate transcripts for SIRT4, SIRT 5 and SIRT6. Given that sirtuins regulate genes involved in several metabolic pathways, oxidative stress responses and cellular stress-induced inflammation^81^, it could be examined whether unbalanced sirtuin signalling contributes to Cre/CreERT toxicity in the cardiovascular system. Such future work is pertinent, because the sirtuin balance regulates DNA repair, whereby SIRT6 activates pathways for high fidelity DNA repair, but SIRT1 promotes DNA repair pathways that have less fidelity and also derepresses p53 transcriptional activity^82^. Moreover, deregulated sirtuin expression might affect cardiovascular function, because SIRT1 regulates gene expression for physiological angiogenesis and activates endothelial nitric oxide synthase for normal vascular function^83,84^.

Together, these findings suggest that further work is required to understand how Cre/ CreER-induced DNA damage is linked to impaired PKA signalling, metabolic effects, inflammation and vascular regulation, and whether perturbing such homeostatic regulatory pathways impacts the interpretation of cardiovascular studies using Cre-LoxP models.

### Genetic dysfunction due to transgene insertion

Cre or CreER expression can be driven from a cassette knocked into an endogenous locus, although such a knock-in approach may disrupt host gene function^85^. Accordingly, Cre and CreER is often expressed from a transgene. However, transgenes integrate randomly into the genome and thereby might disrupt coding or regulatory sequences^86^. For example, the Tg(Wnt1-cre)11Rth transgene (MGI:2386570) has been shown to integrate into the histone gene *H2afv*, causing dopaminergic neuron loss^85,87^. At present, transgene insertion sites are largely unmapped for Cre and CreER transgenes that are commonly used to study the cardiovascular system, with the notable exception of *Myh6-MerCreMer*, which disrupts the *Acf* locus. Therefore this transgene is known as *Acf* Tg(Myh6-cre/Esr1*)1Jmk^88^. Given that the ACF protein is normally undetectable in the heart^88^, it is unlikely that cardiac defects in *Myh6-MerCreMer* mice are due to *Acf* disruption. The finding that cardiac toxicity in *Myh6-MerCreMer* mice is tamoxifen dose-dependent^37,38^ also argues against the theory of transgene insertion as the underlying cause of toxicity. Moreover, toxicity is seen with the independently generated *Myh6-Cre* transgene, which would have integrated randomly into a different genomic locus. Furthermore, adenoviral Cre expression in primary rat cardiomyocyte induces apoptosis independently of transgene insertion or a floxed target gene^38^. Transgene effects are also an unlikely explanation for endothelial CreER^T2^ toxicity, because *Cdh5*-CreER^T2^ and *Pdgfb*-CreER^T2^ mice have independent transgene integrations, but both have CreER toxicity-induced angiogenesis defects that are tamoxifen-dependent.

A transgene might also carry genes other than Cre into the mouse genome. For example, Tg(Ins2-cre)25Mgn and >300 other transgenes contain a human growth hormone minigene to improve transgene expression^85^. In 2015, a study showed that this minigene was shown to reduce the expression of the endogenous growth hormone-releasing hormone through negative feedback^89^. However, it is not known whether minigenes located within Cre or CreER transgenes affect cardiovascular gene function. Together, prevailing evidence suggests that Cre/CreER toxicity occurs independently of transgene insertion, but we cannot exclude that transgene insertion effects exacerbate toxicity.

## Compounding variables for Cre/CreER toxicity

### Cre and CreER expression levels

Several studies have investigated whether Cre/CreER toxicity correlates with expression levels^57,86^. For instance, *CAG-CreER™* activation causes epithelial atrophy in the stomach, but no obvious toxicity in other organs with lower *CAG-CreER™* expression levels^29^. In addition, the liver also lacked toxicity despite expressing high *CAG-CreER™* levels^29^, possibly because this organ has a high regenerative capacity. Aside from tissue and organ differences, the promoter strength and transgene copy number are expected to affect Cre/CreER expression levels.

The copy number effect is illustrated by a comparison of nestin promoter-based transgenes used to drive Cre or CreER expression. Thus, homozygous Tg(Nes-cre)1Wme/J mice (MGI: 2161775) and tamoxifen-treated Tg(Nes-cre/ERT2)4Kag (MGI:3817325) mice both have microencephaly and hydrocephalus [PMID: 16971543]^25^. Given that these transgenes were generated by random integration, their similar phenotype is unlikely caused by disruption of a shared genomic integration site. Instead, heterozygous Tg(Nes-cre)1Wme/J mice and tamoxifen-treated mice carrying a weakly-expressed Nes-CreER^T2^ transgene do not have microencephaly and hydrocephalus^25^, thereby pointing to increased transgene copy number and therefore higher Cre/CreER expression levels as the determinant of toxicity. In agreement, mice with multiple copies of the T-cell targeting *CD4*- CreER^T2^ transgene Tg(Cd4-cre/ERT2)11Gnri have fewer T-cells than Cd4^tm1(cre/ERT2)Thbu^ knock-in mice with a single *CD4*-CreER^T2^ copy^57^.

Similar observations have been made in the cardiovascular system. *Myh6-Cre* (MGI:2386742), which has a copy number of 6, causes very high Cre expression levels in the heart.^90^ In fact, cardiac Cre levels were found to be almost 8-fold higher in *Myh6-Cre* mice than in mice expressing a single copy iSuRe-Cre transgene that utilises the strong and ubiquitous CAG promoter (MGI:6361135)^90^. Should histological cardiac analysis and function electrocardiograms confirm that iSuRe-Cre technology lacks toxicity for the heart, it may become the method of choice for functional studies of cardiac genes via Cre-LoxP technology. Considering potential copy number effects in endothelial cells, CreER levels in Tg(Cdh5-cre/ERT2)1Rha mice are markedly higher than Cre levels in Tg(Tek-cre)1Ywa mice^90^, although copy number variation has been reported for the latter transgene (see above). Whereas Tg(Cdh5-cre/ERT2)1Rha is present in 5 copies^86^, the independently generated Tg(Cdh5-cre/ERT2)1Yka, which also uses the *Cdh5* promoter, was estimated to contain 10 copies (Y. Kubota, personal communication). It is unknown whether this copy number difference impacts retinal endothelial toxicity.

### Tamoxifen/4-OHT dosage

Previous studies have shown that tamoxifen administration can be toxic for mice^91–96^, and even vehicle administration can have deleterious effects^97^. Accordingly, it is now standard practice to administer tamoxifen or 4-OHT to both CreER-positive and CreER-negative mice carrying floxed target genes (e.g., references ^45,98,99^). However, administering tamoxifen/4-OHT to CreER-negative mice does not control for CreER-activation toxicity, which instead requires an additional control, namely tamoxifen/4-OHT-treated mice expressing CreER but lacking floxed target genes. This type of control allows to correct for phenotypes caused by both toxicity from tamoxifen/4-OHT and CreER activation. Accordingly, our 2020 study reported that tamoxifen- or 4-OHT-treated mice expressing CreER have impaired retinal angiogenesis when compared with similarly treated mice lacking CreER^45^.

It should be considered that tamoxifen is more often administered than 4-OHT, mainly due to tamoxifen’s lower cost, but that tamoxifen is metabolised over a longer time frame. Therefore, using tamoxifen typically requires higher doses to achieve the same level of recombination as with 4-OHT, and tamoxifen also affords less precise control over the period in which recombination occurs^20,100^. Interestingly, several studies found that the severity of CreER toxicity phenotypes correlates with the tamoxifen or 4-OHT dose; for example, CreER^T^-expressing MEF cultures exhibit a 4-OHT dose-dependent growth defect^65^. In the cardiovascular system, increasing the tamoxifen dose from 50 μg to 150 μg exacerbated the vascular defects caused by CreER^T2^ activation^45^ (Fig. 3). Moreover, a single tamoxifen dose of 40 mg/kg body weight caused less toxicity in *Myh6*-MerCreMer mice than 20 mg/kg body weight given daily for 5 days, although both regimes induced similar recombination levels^37^. Therefore, it should be examined how different dosing schedules for 4-OHT or tamoxifen compare with respect to CreER toxicity.

Although the mechanistic link between dose and toxicity has not been formally tested, it is conceivable that higher 4-OHT levels in a cell facilitate more CreER translocation to the cell nucleus. In turn, increased nuclear CreER might increase the probability of off-target cleavage within pseudo LoxP sites to induce DNA damage, possibly to an extent that cannot be sufficiently mitigated by DNA repair. In analogy, multiple tamoxifen/4-OHT injections would be expected to prolong nuclear CreER presence, thereby again increasing the probability of off-target cleavage within pseudo LoxP sites. Accordingly, increasing tamoxifen/4-OHT dosage to optimise gene deletion efficiency for cardiovascular phenotyping needs to be balanced against increased off-target effects caused by excessive CreER activation, whereby tamoxifen/4-OHT levels per dose as well as the frequency and interval of doses all need to be considered.

### Cre versus CreER

Several Cre and CreER transgenes have been attributed with causing toxicity in different organ systems, and some evidence suggests that constitutive Cre is less toxic than activated CreER. For example, constitutive Tg(Vil1-cre)1000Gum was reported to be less toxic than tamoxifen-activated Tg(Vil1-cre/ERT2)23Syr for intestinal epithelial cells^28^. A constitutive endothelial Cre transgene, Tg(Tek-cre)1Ywa had no obvious effect on retinal angiogenesis, whereas two different tamoxifen-activated CreER transgenes, Tg(Cdh5-cre/ERT2)#Ykub and Tg(Pdgfb-icre/ERT2)1Frut, both impaired retinal angiogenesis independently of tamoxifen toxicity or floxed target genes^45^ (see above).

To date, no specific mechanism has been identified that might explain increased CreER toxicity compared with Cre toxicity. Nevertheless, it is conceivable that chronic Cre recombinase activity, due to expression of the constitutively active Cre transgene, induces an adaptive response to low level DNA damage, similar to the adaptation of cancer cells to radiation-induced DNA damage^101^. Vice versa, 4-OHT binding to CreER and the ensuing nuclear translocation of activated CreER induces an acute burst in Cre recombination activity that causes extensive and sudden DNA damage, thereby exacerbating proliferation defects or apoptosis incidence. Alternatively, or additionally, damaged cells might simply be replaced over time by unaffected cells to repair Cre-induced tissue damage, whereby the shorter time frame between CreER activation and tissue analysis might be insufficient to observe cell replacement. Alternatively, the fusion of Cre to the ER domain may increase toxicity by enhancing off-target effects, or nuclear 4-OHT localisation might exacerbate adverse effects of CreER endonuclease activity. Given that most studies include tamoxifen administration to control mice, the latter two possibilities are typically controlled for.

Understanding potential differences between Cre versus CreER toxicity is pertinent, because CreER is used increasingly for postnatal studies to circumvent deleterious effects caused by gene deletion at embryonic stages, or when a given promoter is active in multiple cell types during embryogenesis but becomes more specific postnatally. For example, the *Wt1-Cre* expression signature differs between embryonic and adult stages^102^, and *Alb*-Cre is active in the common embryonic progenitor for hepatocytes and cholangiocytes but in adults is active in hepatocytes only^103^. The improved spatiotemporal specificity of genetic deletion with CreER models therefore must be balanced against potentially increased toxicity when choosing CreER over Cre, with further work being required to investigate such possibilities.

## Methods of reducing Cre and CreER toxicity

For experiments in which Cre or CreER toxicity is found to affect experimental readouts, experimental modifications should be considered to reduce toxicity, such as modulating the tamoxifen or 4-OHT dose or its administration frequency or choosing a different Cre/CreER model. Alternatively, it is possible to include appropriate controls for Cre toxicity to correct experimental data accordingly. These options are discussed in detail below.

### 4-OHT versus tamoxifen

As detailed above, both 4-OHT and tamoxifen can cause CreER toxicity *in vivo*, but it remains unclear whether their toxicity differs. Typically, 4-OHT is administered in lower concentrations than tamoxifen, because tamoxifen requires metabolising to yield 4-OHT as the active compound^20^. Accordingly, 4-OHT has an earlier serum peak than tamoxifen^104^ but is metabolised over a shorter timeframe^20,100^. Together, these different properties affect the time window of recombination, but may also impact CreER toxicity. Further work is needed to address these possibilities.

### Choosing a lower tamoxifen/4-OHT dose

Reducing the tamoxifen or 4-OHT dose is a relatively simple starting point to reduce toxicity in CreER^T2^ models. Notably, concentration and dosing schedules vary widely between different studies (Table 2). For example, Tg(Cdh5-cre/ERT2)1Rha has been activated in adult mice with tamoxifen doses as low as 20 mg/kg and as high as 250 mg/kg^105,106^. Given that the extent of toxicity is proportional to the tamoxifen dose given for both endothelial cells and cardiomyocytes^36,45^, it is a good idea to keep the tamoxifen of 4-OHT dose as low as possible whilst still activating CreER. However, a low dose might become rate-limiting for effective recombination, and this, in turn, would impact experimental results^65^. Therefore, it is advisable to perform a dose-response pilot study that controls for toxicity whilst including a recombination reporter to identify the minimal effective dose to activate CreER effectively. A recombination reporter may also help compare tissues from different animals for similar Cre/CreER activity. Yet, the minimally effective dose for activating recombination reporters would probably need to be exceeded to recombine two floxed alleles to homozygosity.

The induction timeline might also affect the extent of toxicity. For example, activating CreER in endothelial cells on different postnatal days might differentially affect retinal angiogenesis. Accordingly, studies should report the dose/dosing regimen and whether tamoxifen or 4-OHT has been used. After an optimal dose and dosing frequency has been established, and the choice of tamoxifen versus 4-OHT has been considered, subsequent experiments should ensure that recombination of floxed target genes of interest is efficient with the chosen regimen.

### Adapting the dosing schedule to key experimental parameters

Tamoxifen metabolism varies by age and strain of mice and the dosing regimen^20,100^. For example, tamoxifen and its metabolites are cleared more slowly in aged mice compared with young adult mice^20^. Moreover, tamoxifen-induced recombination efficiency varies by the gene or cell types targeted^107^. For example, activating *Rosa26*-CreER^T2^ enables highly effective recombination in multiple tissues, such as the skin, liver, stomach and small intestine, but not in the brain, where CreER protein levels are lower^107^. Sex is usually reported and controlled for in adult studies, but rarely in neonatal studies such as for retinal angiogenesis, although it would be good practice. When we investigated CreER toxicity for retinal angiogenesis, there was no difference between the sexes^45^. We suggest that future studies should always consider variables such as age, sex, strain and target tissue when choosing an appropriate tamoxifen or 4-OHT dose for CreER-based studies.

### Choosing a different Cre transgene

As discussed above, some studies suggest that Cre may cause less toxicity than CreER. However, selecting a Cre rather than CreER transgene may not always help, because mice expressing constitutive Tg(Myh6-cre)2182Mds/J have similar cardiac phenotypes to mice expressing activated A1cfTg(Myh6-cre/Esr1*)1Jmk/J^34–38,73^. Notably, any toxicity differences between Cre and CreER would be compounded by copy number variation or promoter strength of transgenes, because both factors determine overall Cre/CreER expression levels (see above). In agreement with this this idea, CreER activated with the same tamoxifen dose and frequency via the *Cdh5* promoter in Tg(Cdh5-cre/ERT2)#Ykub impaired retinal angiogenesis more than via the *Pdgfb* promoter in Tg(Pdgfb-icre/ERT2)1Frut^45^, and this observation correlates with higher *Pdgfb* than *Cdh5* expression levels in endothelial cells. Using a knock-in strategy to reduce Cre or CreER copy number to one per haploid genome might therefore help to limit toxicity^85^. To circumvent disrupting endogenous gene expression after a knockin, the viral 2A peptide or an internal ribosome entry site (IRES) can be used to drive Cre /CreER expression^108^. In summary, selecting a specific transgene influences overall Cre or CreER expression levels, whereby higher Cre or CreER levels are expected to cause more toxicity but induce more gene deletion, causing a methodological conflict that needs to be considered carefully on a case-by-case basis.

### Choosing appropriate controls

In addition to controlling for tamoxifen or vehicle toxicity, as is commonly done, it is possible to control for Cre/CreER toxicity-induced phenotypes by including Cre- or CreER-positive mice lacking a floxed allele. Before our 2020 *Cdh5*-CreR^T2^ toxicity study^45^, a literature search found that only 10 in 222 studies with *Cdh5*-CreR^T2^ reported using a CreER-positive unfloxed control, whereas other studies either did not use this control or used it without explicitly reporting this.

One strategy to obtain Cre/CreER toxicity controls involves breeding two heterozygously floxed mice to each other, whereby one parent also carries the desired Cre transgene. Such a breeding pair yields Cre/CreER-positive offspring carrying no floxed alleles (control) and Cre/CreER-positive offspring carrying two floxed alleles (homozygous mutant), each at a Mendelian frequency of 1:8. Additionally, this breeding strategy produces large numbers of littermate mice with less desirable genotypes, that is, mice with heterozygous floxed alleles or no Cre/CreER. For the latter reason, most studies to date have instead bred homozygously floxed mice to each other whilst including Cre/CreER in one parent, which yields homozygous mutants at a frequency of 1:2 but lacks a Cre/CreER toxicity control.

To balance obtaining Cre/CreER toxicity controls with generating the desired genotypes at a high frequency, it may be practical to establish two parallel breeding strategies: Firstly, pairing a Cre/CreER-positive and Cre/CreER-negative mouse, both lacking floxed target genes, to identify suitable experimental conditions that eliminate or at least minimise toxicity-dependent phenotypes^45^. Secondly, applying the knowledge gained to exclude or account for toxicity effects when using the offspring of Cre/CreER-positive and Cre/CreER-negative mice with homozygous floxed genes, as is currently standard practice, to investigate the phenotypic consequences of gene deletion^99^.

Notably, different control strategies should be considered for mice of different ages. Littermate controls are often used for pre-weaning mice and without prior knowledge of genotype, and results from several litters are typically pooled for analysis. By contrast, genotyped adult mice can be pooled from different litters for an experiment. These considerations would impact the strategy chosen to control for Cre/CreER toxicity.

### Cre mosaic studies

In mosaic studies, two analogous cell populations either express or do not express Cre/CreER^109^. For example, CreER nuclear localisation can be induced at low concentrations to induce recombination in only a subset of cells that also express fluorescent reporters for identifying cells that have undergone CreER-mediated recombination^110^. If performed in the absence of floxed endogenous genes, comparing reporter-positive with reporter-negative cells would show whether Cre or CreER toxicity impacts the cell phenotype and could be used as an experimental approach to identify protocols that reduce toxicity.

### Emerging technologies

To circumvent Cre/CreER toxicity, virally delivered, self-deleting Cre methods have been created, but have not yet been applied to cardiovascular studies and might only be suitable in specific circumstances. Therefore, it has been proposed that a self-deleting Cre, which itself is flanked by LoxP sites, might limit toxicity by restricting its own activity temporally^74^. This approach yielded a recombination frequency similar to CreER activation but lacked a toxicity phenotype *in vitro*^74^ and agrees with the finding that reduced duration of CreER activity has less toxicity (see below). However, viral techniques have pitfalls *in vivo* pitfalls. For example, the self-deleting lentiviral Cre was effectively delivered to cells in the liver and brain, however, some Cre expression occurred also in uninjected liver lobes^74^. Furthermore, viral transduction may cause toxicity, independently of Cre activity; for example, adenoviral methods to introduce Cre caused carcinomas in mice^111^. Finally, not all tissues are equally accessible to viral Cre delivery; for example, this approach might be poorly suitable for studies of early cardiovascular development *in utero*.

The latest evidence suggests that single copy number transgenes such as iSuRe-Cre allow for efficient reporter expression and gene deletion but could also limit Cre toxicity^90^. Further work is therefore warranted to examine whether iSuRe-Cre lacks toxicity in all cell types. Alternatively, Dre-Rox or Crispr/Cas9^112,113^ might circumvent Cre toxicity, although could have other types of off-target effects. Together, the above considerations increase interest in emerging recombination technologies as promising approaches to limit Cre/CreER toxicity in the cardiovascular system.

## Conclusion

Although reports of Cre/CreER toxicity remain scattered in the literature, it is becoming increasingly evident that both constitutive Cre and inducible CreER can negatively affect the health of mammalian cells. Moreover, the breadth of cell types already reported as affected suggests that Cre/CreER toxicity exists in most, if not all mammalian cell types. However, the impact of Cre/CreER toxicity on the interpretation of cardiovascular studies is only beginning to be appreciated, with a handful of reports demonstrating that Cre toxicity can impair angiogenesis, deplete blood cell numbers, cause heart failure and promote glucose intolerance. As the Cre-LoxP system continues to provide a key tool for cardiovascular research, we propose that increasing the use of adequate controls will identify and account for Cre or CreER toxicity and allow investigators to identify optimal experimental parameters that enable efficient gene deletion with minimal toxicity. Given that few cardiovascular studies to date have included controls that protect against the inadvertent reporting of Cre/CreER toxicity-induced phenotypes, we wish to highlight the importance of investigating, understanding, eliminating and controlling for Cre/CreER toxicity in each experimental model. Ultimately, a more widespread approach of this rationale will ensure that cardiovascular studies will report only true phenotypes caused by the Cre/CreER-induced deletion of specific genes of interest.

## Figures and Tables

**Table 1:** Signalling pathways implicated in Cre toxicity

Pathway/Process	Components	Cre model	Refs
**cAMP/PKA**	Phosphorylated CREB, PKIα	Cre (*in vitro)*	77
**DNA damage**	PARP, BAX, γH2AX	*Myh6–Cre*	34
**Inflammation**	iNOS, TGFα1, TNFα, IL1α, IL1α, IL6,	*Amh–Cre*	70
		*Myh6–Cre*	34
**Lipid metabolism**	ABCD3	*Amh–Cre*	70
**NRF2**	NRF2, HO1	*Amh–Cre*	70
**P53/apoptosis**	P53, cleaved caspase 3, PARP, BAX	*Fabp4–Cre*	71
		*Myh6*–Cre	34
**Peroxisome biogenesis**	PEX5, PEX14	*Amh–Cre*	70
**PI3Kα**	PI3Kα	*Myh6*–Cre	36
**PPARa peroxisome metabolism**	MFP1, thiolase B	*Amh–Cre*	70
**Pro-fibrotic**	Col1α1, CTGF	*Myh6*–Cre	34
**ROS metabolism**	Catalase, SOD2, HO1	*Amh–Cre*	70

ABCD3, ATP-binding cassette sub-family D member 3; BAX, BCL2-associated X protein; CREB, cAMP response element-binding protein; Col1α1, collagen alpha-l(I) chain; CTGF, connective tissue growth factor; HO1, heme oxygenase 1; iNOS, inducible NO synthase; MFP1, MAR-binding filament-like protein 1; NFR2, nuclear factor erythroid 2-related factor 2; PI3Kα, phosphatidylinositol 3-kinase regulatory subunit α; PKIα, protein kinase inhibitor α; PARP, poly[ADP-ribose] polymerase; parkin; PEX, peroxisome biogenesis factor; PPARα, peroxisome proliferator-activated receptor α; ROS, reactive oxygen specie; SOD2, superoxide dismutase; TGFβ1, transforming growth factor β1; TNFα, tumour necrosis factor TNF; γH2AX, Ser-139 phosphorylated form of the histone variant H2AX.

**Table 2:** Tamoxifen dosage and toxicity in cardiovascular and hematopoietic studies.

Dosing schedule	Cre model	Toxic effect	Ref
50, 100 or 150 μg tamoxifen in peanut oil Intraperitoneal injection on P2 and P4	*Cdh5*–CreER^T2^	Retinal angiogenesis defects	45
50, 100 or 150 μg tamoxifen in peanut oil Intraperitoneal injection on P2 and P4	*Pdgfb–CreER^T2^*	Retinal angiogenesis defects	45
20 mg/kg/day tamoxifen in soybean oil Intraperitoneal injection on 5 days in adults	*Myh6–merCremer*	Decreased cardiac ejection fraction	37
40 mg/kg/day tamoxifen in soybean oil Intraperitoneal injection on 1 day in adults	*Myh6–merCremer*	None observed	37
40 mg/kg/day tamoxifen in corn oil Oral gavage for 4 days in adults	*Myh6–merCremer*	None observed	36
60 mg/kg/day tamoxifen in corn oil Oral gavage on 4 days in adults	*Myh6–merCremer*	Decreased cardiac ejection fraction Left ventricular dilation	36
60 mg/kg/day tamoxifen in sunflower oil Intraperitoneal injection 3 doses in adults	*Myh6–merCremer*	Decreased cardiac ejection fraction Increased cardiac fibrosis Fractional shortening	38
75 mg/kg/day tamoxifen in corn oil Intraperitoneal injection of pregnant dam on E10.5	CAG–CreER™	Decreased embryonic viability	14
150 mg/kg/day tamoxifen in 90% sunflower oil and 10% ethanol Oral gavage of pregnant dams on E13.5 and E14.5	*Rosa26*–CreER^T2^	Severe anaemia in embryos	55
150 mg/kg/day tamoxifen in 90% sunflower oil and 10% ethanol Oral gavage on 5 days in adults	*Rosa26*–CreER^T2^	Thymic atrophy Increased thymic apoptosis	55

E, embryonic day; P, postnatal day.

## References

[1] Orban PC, Chui D, Marth JD (1992). Tissue- and site-specific DNA recombination in transgenic mice. Proc Natl Acad Sci U S A.

[2] Nagy A (2000). Cre recombinase: the universal reagent for genome tailoring. Genesis.

[3] Agah R (1997). Gene recombination in postmitotic cells. Targeted expression of Cre recombinase provokes cardiac-restricted, site-specific rearrangement in adult ventricular muscle in vivo. J Clin Invest.

[4] Schmidt A (1998). lacZ transgenic mice to monitor gene expression in embryo and adult. Brain research Brain research protocols.

[5] Okabe M, Ikawa M, Kominami K, Nakanishi T, Nishimune Y (1997). ‘Green mice’ as a source of ubiquitous green cells. FEBS Lett.

[6] Li S (2018). Overview of the reporter genes and reporter mouse models. Animal models and experimental medicine.

[7] Muzumdar MD, Tasic B, Miyamichi K, Li L, Luo L (2007). A global double-fluorescent Cre reporter mouse. Genesis.

[8] Madisen L (2010). A robust and high-throughput Cre reporting and characterization system for the whole mouse brain. Nat Neurosci.

[9] Chen CM, Krohn J, Bhattacharya S, Davies B (2011). A comparison of exogenous promoter activity at the ROSA26 locus using a PhiiC31 integrase mediated cassette exchange approach in mouse ES cells. PLoS One.

[10] Kawamoto S (2000). A novel reporter mouse strain that expresses enhanced green fluorescent protein upon Cre-mediated recombination. FEBS Lett.

[11] Sakai K, Miyazaki J (1997). A transgenic mouse line that retains Cre recombinase activity in mature oocytes irrespective of the cre transgene transmission. Biochemical and biophysical research communications.

[12] Heffner CS (2012). Supporting conditional mouse mutagenesis with a comprehensive cre characterization resource. Nat Commun.

[13] Alva JA (2006). VE-Cadherin-Cre-recombinase transgenic mouse: a tool for lineage analysis and gene deletion in endothelial cells. Dev Dyn.

[14] Hayashi S, McMahon AP (2002). Efficient recombination in diverse tissues by a tamoxifen-inducible form of Cre: a tool for temporally regulated gene activation/inactivation in the mouse. Dev Biol.

[15] Feil R (1996). Ligand-activated site-specific recombination in mice. Proc Natl Acad Sci U S A.

[16] Metzger D, Clifford J, Chiba H, Chambon P (1995). Conditional site-specific recombination in mammalian cells using a ligand-dependent chimeric Cre recombinase. Proc Natl Acad Sci U S A.

[17] Feil R, Wagner J, Metzger D, Chambon P (1997). Regulation of Cre recombinase activity by mutated estrogen receptor ligand-binding domains. Biochemical and biophysical research communications.

[18] Littlewood TD, Hancock DC, Danielian PS, Parker MG, Evan GI (1995). A modified oestrogen receptor ligand-binding domain as an improved switch for the regulation of heterologous proteins. Nucleic Acids Res.

[19] Zhang Y (1996). Inducible site-directed recombination in mouse embryonic stem cells. Nucleic Acids Res.

[20] Valny M, Honsa P, Kirdajova D, Kamenik Z, Anderova M (2016). Tamoxifen in the Mouse Brain: Implications for Fate-Mapping Studies Using the Tamoxifen-Inducible Cre-loxP System. Frontiers in cellular neuroscience.

[21] Zhong Q (2015). Boronic prodrug of 4-hydroxytamoxifen is more efficacious than tamoxifen with enhanced bioavailability independent of CYP2D6 status. BMC cancer.

[22] Thanos A (2012). Evidence for baseline retinal pigment epithelium pathology in the Trp1-Cre mouse. Am J Pathol.

[23] Lam PT (2019). Considerations for the use of Cre recombinase for conditional gene deletion in the mouse lens. Hum Genomics.

[24] Amin SR (2019). Viral vector-mediated Cre recombinase expression in substantia nigra induces lesions of the nigrostriatal pathway associated with perturbations of dopamine-related behaviors and hallmarks of programmed cell death. J Neurochem.

[25] Forni PE (2006). High levels of Cre expression in neuronal progenitors cause defects in brain development leading to microencephaly and hydrocephaly. J Neurosci.

[26] Jeannotte L (2011). Unsuspected effects of a lung-specific Cre deleter mouse line. Genesis.

[27] Balkawade RS (2019). Podocyte-specific expression of Cre recombinase promotes glomerular basement membrane thickening. Am J Physiol Renal Physiol.

[28] Bohin N, Carlson EA, Samuelson LC (2018). Genome Toxicity and Impaired Stem Cell Function after Conditional Activation of CreER(T2) in the Intestine. Stem Cell Reports.

[29] Huh WJ, Mysorekar IU, Mills JC (2010). Inducible activation of Cre recombinase in adult mice causes gastric epithelial atrophy, metaplasia, and regenerative changes in the absence of “floxed” alleles. Am J Physiol Gastrointest Liver Physiol.

[30] Li Y, Choi PS, Casey SC, Felsher DW (2014). Activation of Cre recombinase alone can induce complete tumor regression. PLoS One.

[31] Janbandhu VC, Moik D, Fassler R (2014). Cre recombinase induces DNA damage and tetraploidy in the absence of loxP sites. Cell Cycle.

[32] Ng WA, Grupp IL, Subramaniam A, Robbins J (1991). Cardiac myosin heavy chain mRNA expression and myocardial function in the mouse heart. Circ Res.

[33] Sohal DS (2001). Temporally regulated and tissue-specific gene manipulations in the adult and embryonic heart using a tamoxifen-inducible Cre protein. Circ Res.

[34] Pugach EK, Richmond PA, Azofeifa JG, Dowell RD, Leinwand LA (2015). Prolonged Cre expression driven by the alpha-myosin heavy chain promoter can be cardiotoxic. J Mol Cell Cardiol.

[35] Garbern J (2019). Analysis of Cre-mediated genetic deletion of Gdf11 in cardiomyocytes of young mice. Am J Physiol Heart Circ Physiol.

[36] McLean BA (2015). PI3Kalpha is essential for the recovery from Cre/tamoxifen cardiotoxicity and in myocardial insulin signalling but is not required for normal myocardial contractility in the adult heart. Cardiovasc Res.

[37] Lexow J, Poggioli T, Sarathchandra P, Santini MP, Rosenthal N (2013). Cardiac fibrosis in mice expressing an inducible myocardial-specific Cre driver. Dis Model Mech.

[38] Bersell K (2013). Moderate and high amounts of tamoxifen in alphaMHC-MerCreMer mice induce a DNA damage response, leading to heart failure and death. Dis Model Mech.

[39] Kisanuki YY (2001). Tie2-Cre transgenic mice: a new model for endothelial cell-lineage analysis in vivo. Dev Biol.

[40] Theis M (2001). Endothelium-specific replacement of the connexin43 coding region by a lacZ reporter gene. Genesis.

[41] Sorensen I, Adams RH, Gossler A (2009). DLL1-mediated Notch activation regulates endothelial identity in mouse fetal arteries. Blood.

[42] Okabe K (2014). Neurons limit angiogenesis by titrating VEGF in retina. Cell.

[43] Claxton S (2008). Efficient, inducible Cre-recombinase activation in vascular endothelium. Genesis.

[44] Payne S, Val SD, Neal A (2018). Endothelial-Specific Cre Mouse Models. Arteriosclerosis, Thrombosis, and Vascular Biology.

[45] Brash J, Bolton R, Rashbrook V, Denti L, Ruhrberg C (2020). Tamoxifen-activated Cre recombinase impairs angiogenesis independently of gene deletion. Circulation Research.

[46] Kanki Y (2022). Bivalent-histone-marked immediate-early gene regulation is vital for VEGF-responsive angiogenesis. Cell Rep.

[47] Ruhrberg C, Bautch VL (2013). Neurovascular development and links to disease. Cell Mol Life Sci.

[48] Pitulescu ME, Schmidt I, Benedito R, Adams RH (2010). Inducible gene targeting in the neonatal vasculature and analysis of retinal angiogenesis in mice. Nature protocols.

[49] Powner MB (2012). Visualization of gene expression in whole mouse retina by in situ hybridization. Nature protocols.

[50] Toullec A (2018). HIF-1alpha Deletion in the Endothelium, but Not in the Epithelium, Protects From Radiation-Induced Enteritis. Cell Mol Gastroenterol Hepatol.

[51] Mohamed R (2018). Inducible overexpression of endothelial proNGF as a mouse model to study microvascular dysfunction. Biochim Biophys Acta Mol Basis Dis.

[52] Naldini A, Carraro F (2005). Role of inflammatory mediators in angiogenesis. Curr Drug Targets Inflamm Allergy.

[53] Limbourg A (2015). MAP-Kinase Activated Protein Kinase 2 Links Endothelial Activation and Monocyte/macrophage Recruitment in Arteriogenesis. PLoS One.

[54] Ruparelia N, Chai JT, Fisher EA, Choudhury RP (2017). Inflammatory processes in cardiovascular disease: a route to targeted therapies. Nat Rev Cardiol.

[55] Higashi AY (2009). Direct hematological toxicity and illegitimate chromosomal recombination caused by the systemic activation of CreERT2. J Immunol.

[56] Kurachi M, Ngiow SF, Kurachi J, Chen Z, Wherry EJ (2019). Hidden Caveat of Inducible Cre Recombinase. Immunity.

[57] Zeitrag J, Alterauge D, Dahlstrom F, Baumjohann D (2020). Gene dose matters: Considerations for the use of inducible CD4-CreER(T2) mouse lines. European journal of immunology.

[58] Popov D (2010). Endothelial cell dysfunction in hyperglycemia: Phenotypic change, intracellular signaling modification, ultrastructural alteration, and potential clinical outcomes. International Journal of Diabetes Mellitus.

[59] Sweet IR (2009). Endothelial inflammation induced by excess glucose is associated with cytosolic glucose 6-phosphate but not increased mitochondrial respiration. Diabetologia.

[60] Hempel A (1997). High glucose concentrations increase endothelial cell permeability via activation of protein kinase C alpha. Circ Res.

[61] Karbach S (2012). Hyperglycemia and oxidative stress in cultured endothelial cells--a comparison of primary endothelial cells with an immortalized endothelial cell line. J Diabetes Complications.

[62] Duvillie B (1997). Phenotypic alterations in insulin-deficient mutant mice. Proc Natl Acad Sci U S A.

[63] Lee JY (2006). RIP-Cre revisited, evidence for impairments of pancreatic beta-cell function. J Biol Chem.

[64] Pomplun D, Florian S, Schulz T, Pfeiffer AF, Ristow M (2007). Alterations of pancreatic beta-cell mass and islet number due to Ins2-controlled expression of Cre recombinase: RIP-Cre revisited; part 2. Hormone and metabolic research = Hormon- und Stoffwechselforschung = Hormones et metabolisme.

[65] Loonstra A (2001). Growth inhibition and DNA damage induced by Cre recombinase in mammalian cells. Proc Natl Acad Sci U S A.

[66] Silver DP, Livingston DM (2001). Self-excising retroviral vectors encoding the Cre recombinase overcome Cre-mediated cellular toxicity. Mol Cell.

[67] Thyagarajan B, Guimaraes MJ, Groth AC, Calos MP (2000). Mammalian genomes contain active recombinase recognition sites. Gene.

[68] Semprini S (2007). Cryptic loxP sites in mammalian genomes: genome-wide distribution and relevance for the efficiency of BAC/PAC recombineering techniques. Nucleic Acids Res.

[69] Norbury CJ, Zhivotovsky B (2004). DNA damage-induced apoptosis. Oncogene.

[70] Xiao Y (2012). Cre-mediated stress affects sirtuin expression levels, peroxisome biogenesis and metabolism, antioxidant and proinflammatory signaling pathways. PLoS One.

[71] Zhu J, Nguyen MT, Nakamura E, Yang J, Mackem S (2012). Cre-mediated recombination can induce apoptosis in vivo by activating the p53 DNA damage-induced pathway. Genesis.

[72] Wang X, Lauth A, Wan TC, Lough JW, Auchampach JA (2020). Myh6-driven Cre recombinase activates the DNA damage response and the cell cycle in the myocardium in the absence of loxP sites. Dis Model Mech.

[73] Rehmani T, Salih M, Tuana BS (2019). Cardiac-Specific Cre Induces Age-Dependent Dilated Cardiomyopathy (DCM) in Mice. Molecules.

[74] Pfeifer A, Brandon EP, Kootstra N, Gage FH, Verma IM (2001). Delivery of the Cre recombinase by a self-deleting lentiviral vector: efficient gene targeting in vivo. Proc Natl Acad Sci U S A.

[75] Baba Y, Nakano M, Yamada Y, Saito I, Kanegae Y (2005). Practical range of effective dose for Cre recombinase-expressing recombinant adenovirus without cell toxicity in mammalian cells. Microbiol Immunol.

[76] Naiche LA, Papaioannou VE (2007). Cre activity causes widespread apoptosis and lethal anemia during embryonic development. Genesis.

[77] Gangoda L (2012). Cre transgene results in global attenuation of the cAMP/PKA pathway. Cell Death Dis.

[78] Sassone-Corsi P (2012). The cyclic AMP pathway. Cold Spring Harb Perspect Biol.

[79] Pepin G (2016). Cre-dependent DNA recombination activates a STING-dependent innate immune response. Nucleic Acids Res.

[80] Gerhart-Hines Z (2011). The cAMP/PKA pathway rapidly activates SIRT1 to promote fatty acid oxidation independently of changes in NAD(+). Mol Cell.

[81] Merksamer PI (2013). The sirtuins, oxidative stress and aging: an emerging link. Aging (Albany NY).

[82] Roth M, Chen WY (2014). Sorting out functions of sirtuins in cancer. Oncogene.

[83] Potente M (2007). SIRT1 controls endothelial angiogenic functions during vascular growth. Genes & development.

[84] Kitada M, Ogura Y, Koya D (2016). The protective role of Sirt1 in vascular tissue: its relationship to vascular aging and atherosclerosis. Aging (Albany NY).

[85] Goodwin LO (2019). Large-scale discovery of mouse transgenic integration sites reveals frequent structural variation and insertional mutagenesis. Genome Res.

[86] Cain-Hom C (2017). Efficient mapping of transgene integration sites and local structural changes in Cre transgenic mice using targeted locus amplification. Nucleic Acids Res.

[87] Lewis AE, Vasudevan HN, O’Neill AK, Soriano P, Bush JO (2013). The widely used Wnt1-Cre transgene causes developmental phenotypes by ectopic activation of Wnt signaling. Dev Biol.

[88] Harkins S, Whitton JL (2016). Chromosomal mapping of the alphaMHC-MerCreMer transgene in mice reveals a large genomic deletion. Transgenic research.

[89] Declercq J (2015). Metabolic and Behavioural Phenotypes in Nestin-Cre Mice Are Caused by Hypothalamic Expression of Human Growth Hormone. PLoS One.

[90] Fernandez-Chacon M (2019). iSuRe-Cre is a genetic tool to reliably induce and report Cre-dependent genetic modifications. Nat Commun.

[91] Ved N, Curran A, Ashcroft FM, Sparrow DB (2019). Tamoxifen administration in pregnant mice can be deleterious to both mother and embryo. Laboratory animals.

[92] Wyatt KD, Sakamoto K, Watford WT (2021). Tamoxifen administration induces histopathologic changes within the lungs of Cre-recombinase-negative mice: A case report. Laboratory animals.

[93] Keeley TM, Horita N, Samuelson LC (2019). Tamoxifen-Induced Gastric Injury: Effects of Dose and Method of Administration. Cell Mol Gastroenterol Hepatol.

[94] Patel SH (2017). Low-dose tamoxifen treatment in juvenile males has long-term adverse effects on the reproductive system: implications for inducible transgenics. Sci Rep.

[95] Sun MR, Steward AC, Sweet EA, Martin AA, Lipinski RJ (2021). Developmental malformations resulting from high-dose maternal tamoxifen exposure in the mouse. PLoS One.

[96] Ahn SH (2019). Tamoxifen suppresses pancreatic beta-cell proliferation in mice. PLoS One.

[97] Alsina-Sanchis E (2021). Intraperitoneal Oil Application Causes Local Inflammation with Depletion of Resident Peritoneal Macrophages. Mol Cancer Res.

[98] Brash JT, Denti L, Ruhrberg C, Bucher F (2019). VEGF188 promotes corneal reinnervation after injury. JCI insight.

[99] Fantin A (2013). NRP1 acts cell autonomously in endothelium to promote tip cell function during sprouting angiogenesis. Blood.

[100] Jahn HM (2018). Refined protocols of tamoxifen injection for inducible DNA recombination in mouse astroglia. Sci Rep.

[101] Carlos-Reyes A, Muniz-Lino MA, Romero-Garcia S, Lopez-Camarillo C, Hernandez-de la Cruz ON (2021). Biological Adaptations of Tumor Cells to Radiation Therapy. Front Oncol.

[102] Wilm TP (2021). Restricted differentiative capacity of Wt1-expressing peritoneal mesothelium in postnatal and adult mice. Sci Rep.

[103] Zorn AM, Wells JM (2009). Vertebrate endoderm development and organ formation. Annu Rev Cell Dev Biol.

[104] Stearns V (2003). Active tamoxifen metabolite plasma concentrations after coadministration of tamoxifen and the selective serotonin reuptake inhibitor paroxetine. J Natl Cancer Inst.

[105] Fan Q, Mao H, Xie L, Pi X (2019). Prolyl Hydroxylase Domain-2 Protein Regulates Lipopolysaccharide-Induced Vascular Inflammation. Am J Pathol.

[106] Ding BS (2011). Endothelial-derived angiocrine signals induce and sustain regenerative lung alveolarization. Cell.

[107] Hameyer D (2007). Toxicity of ligand-dependent Cre recombinases and generation of a conditional Cre deleter mouse allowing mosaic recombination in peripheral tissues. Physiol Genomics.

[108] Kim JH (2011). High cleavage efficiency of a 2A peptide derived from porcine teschovirus-1 in human cell lines, zebrafish and mice. PLoS One.

[109] Jakobsson L (2010). Endothelial cells dynamically compete for the tip cell position during angiogenic sprouting. Nat Cell Biol.

[110] Luo W (2021). Arterialization requires the timely suppression of cell growth. Nature.

[111] Meuwissen R, Linn SC, van der Valk M, Mooi WJ, Berns A (2001). Mouse model for lung tumorigenesis through Cre/lox controlled sporadic activation of the K-Ras oncogene. Oncogene.

[112] Han X (2021). A suite of new Dre recombinase drivers markedly expands the ability to perform intersectional genetic targeting. Cell stem cell.

[113] Tycko J (2019). Mitigation of off-target toxicity in CRISPR-Cas9 screens for essential noncoding elements. Nat Commun.

